# Protonation of Enniatin B in Nitrobenzene: Experimental and Theoretical Study with Respect to Other Univalent Cations

**DOI:** 10.1021/acsomega.5c02470

**Published:** 2025-09-17

**Authors:** Petr Vaňura, Stanislav Böhm, Tereza Uhlíková

**Affiliations:** 52735 University of Chemistry and Technology, Prague, Technická 5, 166 28 Prague 6, Czech Republic

## Abstract

Based on extraction experiments, the exchange extraction constant of the reaction H_3_O^+^(aq) + **1·**Na^+^(nb) ⇄ **1·**H_3_O^+^(nb) + Na^+^(aq) in the system water–nitrobenzene (**1** = enniatin B; aq = aqueous phase, nb = nitrobenzene phase) was evaluated to be log *K*_ex_ (H_3_O^+^, **1·**Na^+^) = 0.9 ± 0.15. Furthermore, the stability constant of the **1·**H_3_O^+^ complex in water-saturated nitrobenzene was calculated for a temperature of 25 °C as log *K*_nb_ (**1·**H_3_O^+^) = 6.4 ± 0.2. Analogously, the stability constants of some other univalent metal cations have been determined by a similar method. Additionally, quantum mechanical calculations (M06-D3/DefSVPP in nitrobenzene) were used to determine the most probable structures of the cationic complexes **1·**H_3_O^+^ and **1·**Na^+^. For comparison, three different conformers of **1·**H_3_O^+^ (nb) were also investigated, considering the nesting H_3_O^+^ ion into the enniatin B ring. In the most stable resulting complex, the H_3_O^+^ cation is centrally positioned and stabilized by three relatively strong hydrogen bonds to the three carbonyl oxygen atoms of the parent enniatin B ligand. The interaction energies, *E*(int), of the **1·**H_3_O^+^ and **1·**Na^+^ complexes in nitrobenzene were found to be 196.81 and 82.19 kJ/mol, respectively, confirming the formation of protonated complexes.

## Graphical abstract

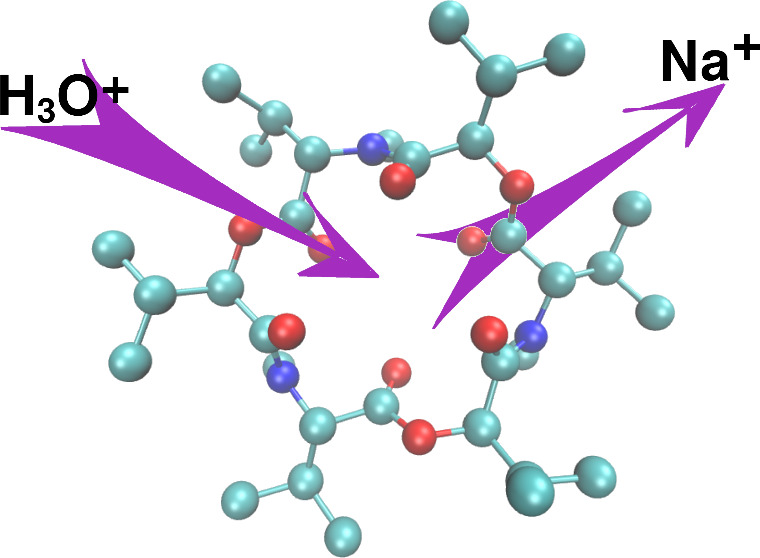

## 1 Introduction

Enniatins are a class of cyclohexadepsipeptide mycotoxins produced by *Gnomonia errabunda* and various *Fusarium* species. They exhibit a range of biological activities, such as phytotoxic, insecticidal, antimicrobial, and antibiotic. Enniatins also have ionophoric properties.^1–3^ Naturally occurring enniatins are typically found as a mixture of cyclic depsipeptides, predominantly enniatins A, A1, B, and B1, with smaller quantities of variants, such as enniatins C, D, E, and F. Structurally, these cyclic hexadepsipeptides consist of alternating *N*-methylated amino acids and hydroxy acid residues. All known enniatins and also beauvericin share a common internal cavity bordered by six polar carbonyl groups capable of coordinating cations. Their exterior surfaces are slightly different from each other, composed of isopropyl and methyl groups. These groups enable the molecule or its cation complex to dissolve in organic solvents.^4^ Enniatins can integrate into cell membranes, where they form cation-selective pores. These pores facilitate the transport of monovalent ions, particularly across mitochondrial membranes, affecting oxidative phosphorylation uncoupling^3,5^

Kamyar et al.^6^ investigated enniatin complexes with alkali metal (Li^+^, Na^+^, K^+^) and alkaline earth metal (Mg^2+^, Ca^2+^) cations, ranking their selectivity as follows: K^+^ > Ca^2+^ ≈ Na^+^ > Mg^2+^ > Li^+^. Similarly, Ivanov et al.,^7^ using NMR spectroscopy, demonstrated that enniatin B forms complexes with Na^+^, K^+^, and Cs^+^. A comparative study by Lifson et al.^4^ revealed that, while both enniatin and valinomycin can bind alkali metal cations, valinomycin exhibits greater selectivity.

The complexes of enniatin B with potassium, cesium, and ammonium have been proven by the solvent extraction method, and their structures have been found by quantum mechanical DFT calculations. In each complex, the univalent cation is located in the center of the cavity of the enniatin B molecule.^8–10^ Recently, protonation of valinomycin,^11^ beauvericin,^12^ nonactin,^13^ antamanide,^14^ and a hexaarylbenzene-based receptor^15^ has been investigated in detail. In the beauvericin–H_3_O^+^ complex the three hydrogen atoms of the H_3_O^+^ ion are bonded to the three oxygens in the internal cavity of beauvericin.

It must be pointed out that the stability constants of complex cations can be calculated only in highly polar solvents. In the nonpolar medium only neutral particles (compounds of a complex cation and a respective anion) can exist.

In this study, the liquid–liquid extraction of H_3_O^+^ into nitrobenzene was investigated using a synergistic mixture of sodium dicarbollylcobaltate (NaDCC) and the enniatin B ligand (abbrev. **1**; see Scheme 1). The stability constant of the identified **1·**H_3_O^+^ complex in the organic phase of the extraction system water–nitrobenzene has been experimentally determined and compared with those of other ions. Furthermore, quantum mechanical calculations were employed to perform a conformational analysis, leading to identification of the most probable structure of this cationic complex. Finally, the interaction energies between ligand and ions (H_3_O^+^, Na^+^) in different solutions were calculated as well. Given the importance of enniatin B in biochemistry and its potential use as an ion carrier, these findings represent a significant contribution to the host–guest chemistry of this natural ionophore.

**Scheme 1:**
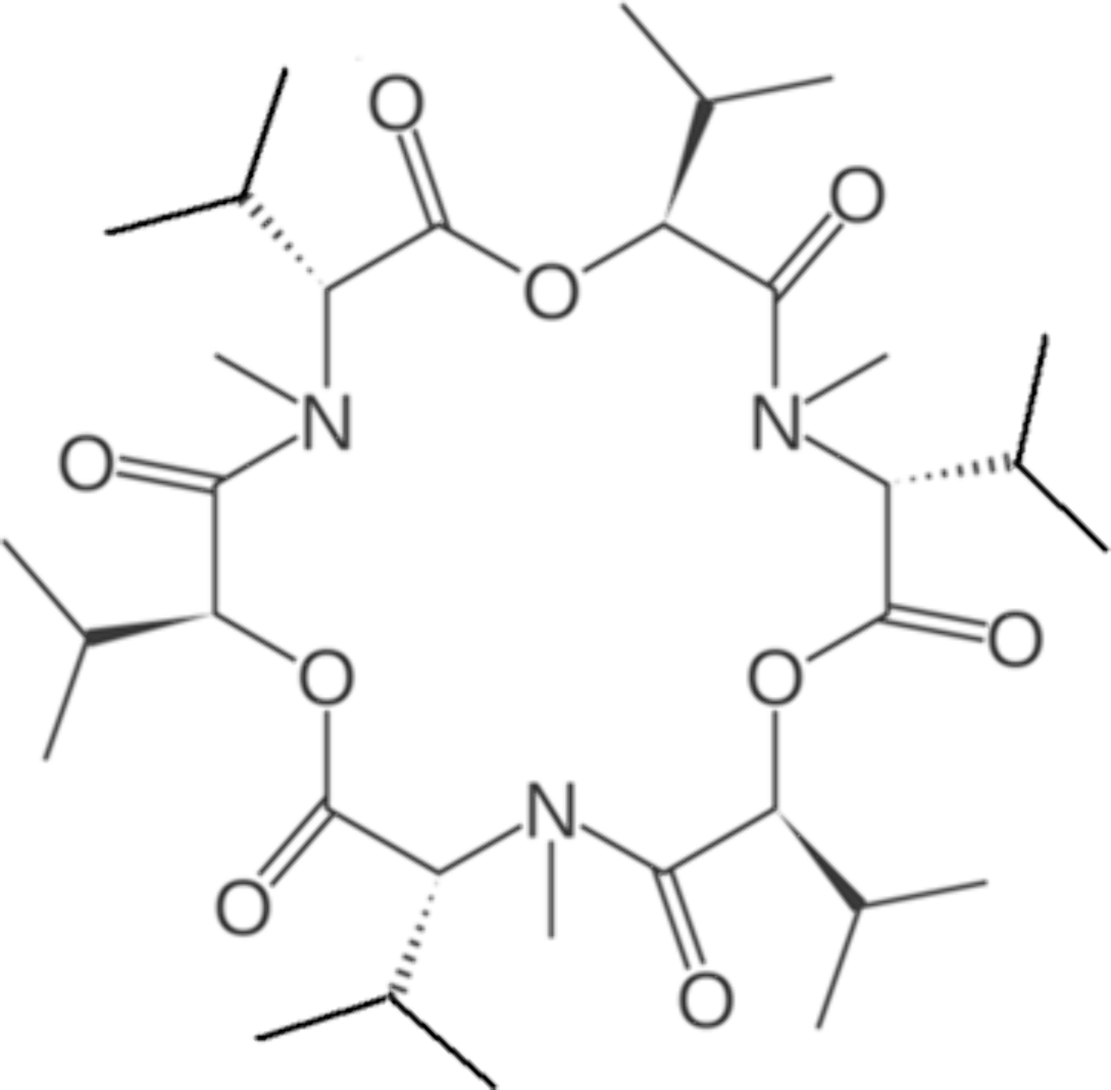
Structural Formula of Enniatin B (Abbrev. **1**)

## 2 Experimental Section

Enniatin B (puriss., ≥99%; see Scheme 1) was purchased from Aldrich, and it was employed as received. Cesium dicarbollylcobaltate, CsDCC, was synthesized by the method of Hawthorne et al.^16^ The other chemicals used (Lachema, Brno, Czech Republic) were of reagent grade purity. A nitrobenzene solution of sodium dicarbollylcobaltate (NaDCC) was prepared from CsDCC by the procedure described elsewhere.^17^ An aqueous solution of sodium picrate (abbrev. NaA) was prepared by dissolving the known weight of picric acid in a stoichiometric amount of an aqueous solution of NaOH. DuPont, Belgium delivered the radionuclide ^22^Na^+^; its radionuclidic purity was 99.9%.

Aqueous solutions of sodium picrate, the concentration of which varied from 0.00025 to 0.001 M and 10 kBq of ^22^Na^+^, were extracted by the solutions of enniatin B in nitrobenzene. The concentration of enniatin B in the organic phase, *C*_1_^in,nb^, varied from 0.001 to 0.003 M and was always higher than the concentration of sodium picrate in the aqueous phase, *C*_NaA_^in,aq^.

The protonation constant of enniatin B in nitrobenzene was determined using competitive extraction. Two mL of an aqueous solution of HCl, the concentration of which varied in the range from 1 × 10^–3^ to 5 × 10^–3^ M, and 10 kBq of ^22^Na^+^ were added to 2 mL of a nitrobenzene solution of **1** and NaDCC, whose initial concentration varied also from 1 × 10^–3^ to 5 × 10^–3^ M. In all experiments the initial concentration of HCl in the aqueous phase (*C*_HCl_^in,aq^) was equal to the initial concentration of **1** in nitrobenzene, *C*_1_^in,nb^, and to the initial concentration of NaDCC in this medium, *C*_NaDCC_^in,nb^ (i.e. *C*_HCl_^in,aq^ = *C*_1_^in,nb^ = *C*_NaDCC_^in,nb^).

The extraction experiments were carried out in 10 mL polypropylene test tubes with polypropylene stoppers. Two mL of each phase was shaken for 2 h at 25 ± 1 °C, using a laboratory shaker. Furthermore, the phases were separated by centrifugation (5 min, 3000 rpm), and the γ-activities of 1 mL samples of each phase were measured using a well-type NaI­(Tl) scintillation detector connected to a γ-analyzer Triathler (Hidex, Turku, Finland). The equilibrium distribution ratios of sodium, *D*_Na_, were determined as the ratios of the corresponding measured radioactivities of ^22^Na^+^ in the nitrobenzene and aqueous samples.

## 3 Results and Discussion

### 3.1 Extraction Experiments

Regarding the results of previous papers,^17–19^ the two–phase water–NaA (A^–^ = picrate)–nitrobenzene extraction system can be described by the equilibrium:

1 $${\mathrm{Na}}^{+}\left(\mathrm{aq}\right)+A^{-}\left(\mathrm{aq}\right)⇄{\mathrm{Na}}^{+}\left(\mathrm{nb}\right)+A^{-}\left(\mathrm{nb}\right);K_{\mathrm{ex}}\left({\mathrm{Na}}^{+},A^{-}\right)$$

with the corresponding extraction constant *K*_ex_ (Na^+^, A^–^); “aq” and “nb” denote the presence of the species in the aqueous and nitrobenzene phases, respectively. It must be pointed out that in the highly polar nitrobenzene phase, the extracted salts or acids are fully dissociated. Both cations and anions pass into the organic phase separately, while the conditions of electroneutrality of both phases must be maintained. Therefore, the value of *K*_ex_ (Na^+^, A^–^) can be calculated using the individual extraction constants of sodium cation *K*_Na^+^_^i^ and picrate anion *K*_A^–^_^i^ by means of the equation

2 $$\log K_{\mathrm{ex}}\left({\mathrm{Na}}^{+},A^{-}\right)=\log K_{{\mathrm{Na}}^{+}}^{i}+\log K_{A^{-}}^{i}$$

Using the values log *K*_Na^+^_^i^ = – 6.0 ^19^ and log *K*_A^–^_^i^ = 0.8 (A^–^ = picrate),^19^ the extraction constant *K*_ex_ (Na^+^, A^–^) can be calculated as log *K*_ex_ (Na^+^, A^–^) = −5.2.

The extraction in the two-phase water–NaA (A^–^ = picrate)–nitrobenzene–**1** (enniatin B) system is described by the following main chemical equilibrium:^18^

3 Na+(aq)+A−(aq)+1(nb)⇄1·Na+(nb)+A−(nb);Kex(1·Na+,A−)

The equilibrium extraction constant of reaction 3, *K*_ex_ (**1·**Na^+^, A^–^), can be written as

4 $$K_{\mathrm{ex}}\left(1\cdot Na^{+},A^{-}\right)=\frac{{\left[1\cdot Na^{+}\right]}_{nb}{\left[A^{-}\right]}_{nb}}{{\left[Na^{+}\right]}_{aq}{\left[A^{-}\right]}_{aq}{\left[1\right]}_{nb}}$$

A lipophilic ligand, such as enniatin B, is practically present in the nitrobenzene phase only, where this ligand forms very stable complexes with the Na^+^ and H^+^ cations, as given below.

The distribution ratio of sodium can be calculated by the equation

5 $$D_{\mathrm{Na}}=\frac{{\left[1\cdot Na^{+}\right]}_{nb}+{\left[Na^{+}\right]}_{nb}}{{\left[Na^{+}\right]}_{aq}}$$

For [**1·**Na^+^]_nb_ ≫ [Na^+^]_nb_ (this condition if fulfilled because the extraction of sodium picrate in the absence of ligand is negligible) eq 5 transforms to

6 $$D_{\mathrm{Na}}=\frac{{\left[1\cdot NaL^{+}\right]}_{nb}}{{\left[Na^{+}\right]}_{aq}}=\frac{C_{\mathrm{NaA}}^{\mathrm{eq},\mathrm{nb}}}{C_{\mathrm{NaA}}^{\mathrm{eq},\mathrm{aq}}}$$

where *C*_NaA_^eq,aq^ and *C*_NaA_^eq,nb^ are the equilibrium concentrations of sodium picrate in the aqueous and organic phases.

Applying the mass balances of enniatin B and sodium picrate at equal volumes of the phases, and the conditions of electroneutrality in the aqueous and organic phases ([**1·** Na^+^]_nb_ = [A^–^]_nb_ = *C*_NaA_^eq,nb^ and [Na^+^]_aq_ = [A^–^]_aq_ = *C*_NaA_^eq,aq^), combined with eq 4, we obtain the final expression for the extraction constant *K*_ex_ (**1·**Na^+^, A^–^) in the following form:

7 $$K_{\mathrm{ex}}\left(1\cdot {\mathrm{Na}}^{+},A^{-}\right)=D_{\mathrm{Na}}^{2}/\left\{C_{1}^{\mathrm{in},\mathrm{nb}}-\frac{D_{\mathrm{Na}}}{1+D_{\mathrm{Na}}}C_{\mathrm{NaA}}^{\mathrm{in},\mathrm{aq}}\right\}$$

where *C*_NaA_^in,aq^ is the initial concentration of NaA in the aqueous phase and *C*_1_^in,nb^ denotes the initial concentration of **1** in the organic phase. The results are summarized in Table 1.

**Table 1:** Experimental Data Concerning Determination of log *K*_ex_ (**1·**Na^+^, A^–^) Based on Eq 7

*C*_NaA_^in,aq^ (M)	*C*_**1**_^in,nb^ (M)	*D*_Na_	log *K*_ex_ (**1·**Na^+^, A^–^)
2.5 × 10^–4^	1 × 10^–3^	0.052	0.43
5 × 10^–4^	1 × 10^–3^	0.064	0.63
5 × 10^–4^	2 × 10^–3^	0.104	0.73
5 × 10^–4^	3 × 10^–3^	0.102	0.54
1 × 10^–3^	2 × 10^–3^	0.115	0.82

Using eq 7, log *K*_ex_ (**1·**Na^+^, A^–^) = 0.6 ± 0.1 (five points, see Table 1) has been determined. The results in Table 1 show that the values of log *K*_ex_ (**1·**Na^+^, A^–^) are independent of concentration, which experimentally proves the extraction mechanism, expressed by the two-phase chemical equilibrium (eq 3).

The stability constant of the enniatin B complex in the nitrobenzene phase, i.e. the equilibrium constant of the reaction 8

8 $$1\left(\mathrm{nb}\right)+{\mathrm{Na}}^{+}\left(\mathrm{nb}\right)⇄1\cdot {\mathrm{Na}}^{+}\left(\mathrm{nb}\right)$$

is expressed as

9 $$K_{\mathrm{nb}}\left(1\cdot {\mathrm{Na}}^{+}\right)=\frac{{\left[1\cdot {\mathrm{Na}}^{+}\right]}_{\mathrm{nb}}}{{\left[{\mathrm{Na}}^{+}\right]}_{\mathrm{nb}}{\left[1\right]}_{\mathrm{nb}}}$$

Knowing the extraction constants *K*_ex_ (Na^+^, A^–^) and *K*_ex_ (**1·**Na^+^, A^–^), the stability constant of complex **1·**Na^+^ in nitrobenzene saturated with water, defined by eq 9, can be calculated by eq 10

10 $$\log K_{\mathrm{nb}}\left(1\cdot {\mathrm{Na}}^{+}\right)=\log K_{\mathrm{ex}}\left(1\cdot {\mathrm{Na}}^{+},A^{-}\right)-\log K_{\mathrm{ex}}\left({\mathrm{Na}}^{+},A^{-}\right)$$

Applying eq 10 for the constants log K_ex_ (Na^+^, A^–^) = −5.2 and log K_ex_ (**1·**Na^+^, A^–^) = 0.6 given above, we obtain the stability constant of the **1·**Na^+^ complex in water-saturated nitrobenzene at 25 °C as log *K*_nb_ (**1·**Na^+^) = 5.8 ± 0.15 (Standard deviation, five points).

From the previous papers it can be derived that the two-phase extraction system water–HCl–nitrobenzene–**1** (enniatin B)–sodium dicarbollylcobaltate (NaDCC) is described by the chemical equilibrium:^11–15^

11 $$H_{3}O^{+}\left(\mathrm{aq}\right)+1\cdot {\mathrm{Na}}^{+}\left(\mathrm{nb}\right)⇄1\cdot H_{3}O^{+}\left(\mathrm{nb}\right)+{\mathrm{Na}}^{+}\left(\mathrm{aq}\right);K_{\mathrm{ex}}\left(H_{3}O^{+},1\cdot {\mathrm{Na}}^{+}\right)$$

The equilibrium extraction constant *K*_ex_(H_3_O^+^, **1·**Na^+^) of eq 11 can be described as

12 $$K_{\mathrm{ex}}\left(H_{3}O^{+},1\cdot {\mathrm{Na}}^{+}\right)=\frac{{\left[1\cdot H_{3}O^{+}\right]}_{\mathrm{nb}}{\left[{\mathrm{Na}}^{+}\right]}_{\mathrm{aq}}}{{\left[H_{3}O^{+}\right]}_{\mathrm{aq}}{\left[1\cdot {\mathrm{Na}}^{+}\right]}_{\mathrm{nb}}}$$

If [**1·**Na^+^]_nb_ ≫ [Na^+^]_nb_ and [**1·**H_3_O^+^]_nb_ ≫ [H_3_O^+^]_nb_, i.e. for log *K*_nb_(**1·**Na^+^) > 5 and log *K*_nb_(**1·**H_3_O^+^) > 5, which is fulfilled, see below, the concentration [**1·**H_3_O^+^] is equal to the concentration of extracted H_3_O^+^ cation in nitrobenzene and also [**1·**Na^+^] is equal to the concentration of Na^+^ cation in the organic phase.

Using the conditions of electroneutrality in both phases and the mass balances of Na^+^ and H_3_O^+^ cations studied at equal volumes of the phases, we gain the final expression for *K*_ex_(H_3_O^+^, **1**·Na^+^) in the following form:

13 $$K_{\mathrm{ex}}\left(H_{3}O^{+},1\cdot {\mathrm{Na}}^{+}\right)=\frac{1}{D_{\mathrm{Na}}}\frac{C_{\mathrm{NaDCC}}^{\mathrm{in},\mathrm{nb}}}{\left(1+D_{\mathrm{Na}}\right)C_{\mathrm{HCl}}^{\mathrm{in},\mathrm{aq}}-C_{\mathrm{NaDCC}}^{\mathrm{in},\mathrm{nb}}}$$

where *C*_HCl_^in,aq^ is the initial concentration of HCl in the aqueous phase and *C*_NaDCC_^in,nb^ denotes the initial concentration of NaDCC in the organic phase of the system under consideration.

For *C*_NaDCC_^in,nb^ = *C*_HCl_^in,aq^ = *C*_1_^in,nb^ eq 13 transforms to

14 $$K_{\mathrm{ex}}\left(H_{3}O^{+},1\cdot {\mathrm{Na}}^{+}\right)=\frac{1}{D_{\mathrm{Na}}^{2}}$$

In this work, from the extraction experiments by means of eq 14, the value of the constant *K*_ex_(H_3_O^+^, **1·**Na^+^) was determined as log *K*_ex_(H_3_O^+^, **1·**Na^+^) = 0.9 ± 0.15 (see Table 2). The fact that the calculated values of the stability constants do not show any trend confirms the correctness of the proposed mechanism.

**Table 2:** Experimental Data Concerning Determination of log *K*_ex_ (H_3_O^+^, **1**. Na^+^) Based on Eq 14

*C*_HCl_^in,aq^ (M)	*C*_NaDCC_^in,nb^ (M)	*D*_Na_	log *K*_ex_ (H_3_O^+^, **1**· Na^+^)
1 × 10^–3^	1 × 10^–3^	0.32	1.0
2 × 10^–3^	2 × 10^–3^	0.35	0.9
3 × 10^–3^	3 × 10^–3^	0.33	1.0
4 × 10^–3^	4 × 10^–3^	0.36	0.9
5 × 10^–3^	5 × 10^–3^	0.38	0.8

Our previous results^11–15^ show that the stability constants *K*_nb_(**1·**H_3_O^+^) can be calculated by eq 15 as

15 $$\log K_{\mathrm{nb}}\left(1\cdot H_{3}O^{+}\right)=\log K_{\mathrm{nb}}\left(1\cdot {\mathrm{Na}}^{+}\right)+\log K_{\mathrm{ex}}\left(H_{3}O^{+},1\cdot {\mathrm{Na}}^{+}\right)-\log K_{\mathrm{ex}}\left(H_{3}O^{+},{\mathrm{Na}}^{+}\right)$$

Knowing the value log K_ex_(H_3_O^+^, Na^+^) = 0.3 inferred from ref 19 and the constants log *K*_ex_(H_3_O^+^, **1·**Na^+^) and log *K*_nb_(**1·**Na^+^) given above, and applying eq 15, the stability constant of the **1**·H_3_O^+^ complex in water-saturated nitrobenzene at 25 °C has been calculated as log *K*_nb_(**1·**H_3_O^+^) = 6.4 ± 0.2 (Standard deviation, five points). It can be proven, from the values of log *K*_nb_(**1·**H_3_O^+^) = 6.4 and log *K*_nb_(**1·**Na^+^) = 5.8, that the conditions [**1·**Na^+^]_nb_ ≫ [Na^+^]_nb_ and [**1·**H^+^]_nb_ ≫ [H^+^]_nb_ are fulfilled, so eq 14 is valid.

Table 3 summarizes the protonation constants of some natural ionophores in nitrobenzene saturated with water. It is clear from Table 3 that the protonation constants increase in the sequence beauvericin < valinomycin < antamanide < enniatin B < nonactin.

**Table 3:** Protonation Constants of Natural Ionophores in Nitrobenzene Saturated with Water (L = Ionophore)

Ionophore	log *K*(H_3_OL^+^)	Ref
Enniatin B	6.4	This work
Nonactin	6.6	^13^
Beauvericin	4.4	^12^
Valinomycin	5.3	^11^
Antamanide	5.7	^14^

Furthermore, the stability constants of the complexes **1·**Li^+^, **1·**Rb^+^ and **1·**Tl^+^ have been determined by the same method, as the protonation constant of enniatin B. All these stability constants, along with some data from the literature, are summarized in Table 4 and Figure 1. The stability constants of alkali metal cations decrease in the order Li^+^ > Na^+^ > K^+^ > Rb^+^ > Cs^+^. The dependence of the logarithm of the stability constants on the crystallographic radius of the alkali metal cation is linear (R^2^ = 0.92); see Figure 1. The stability constants of NH_4_^+^ are higher than those of alkali metal cations with the same ionic radius. It must be pointed out that both H_3_O^+^ and NH_4_^+^ ions are bonded to enniatin B via three hydrogen atoms; see below.

**Table 4:** Stability Constants of Several Univalent Cations with Enniatin B in Water-Saturated Nitrobenzene

Ion	log *K*(ML^+^)	Ref
Li^+^	7.4	This work
H_3_O^+^	6.4	This work
Na^+^	5.8	This work
K^+^	5.5	^10^
Rb^+^	4.9	This work
Cs^+^	4.2	^8^
NH_4_^+^	6.4	^9^
Tl^+^	5.2	This work

**Figure 1:**
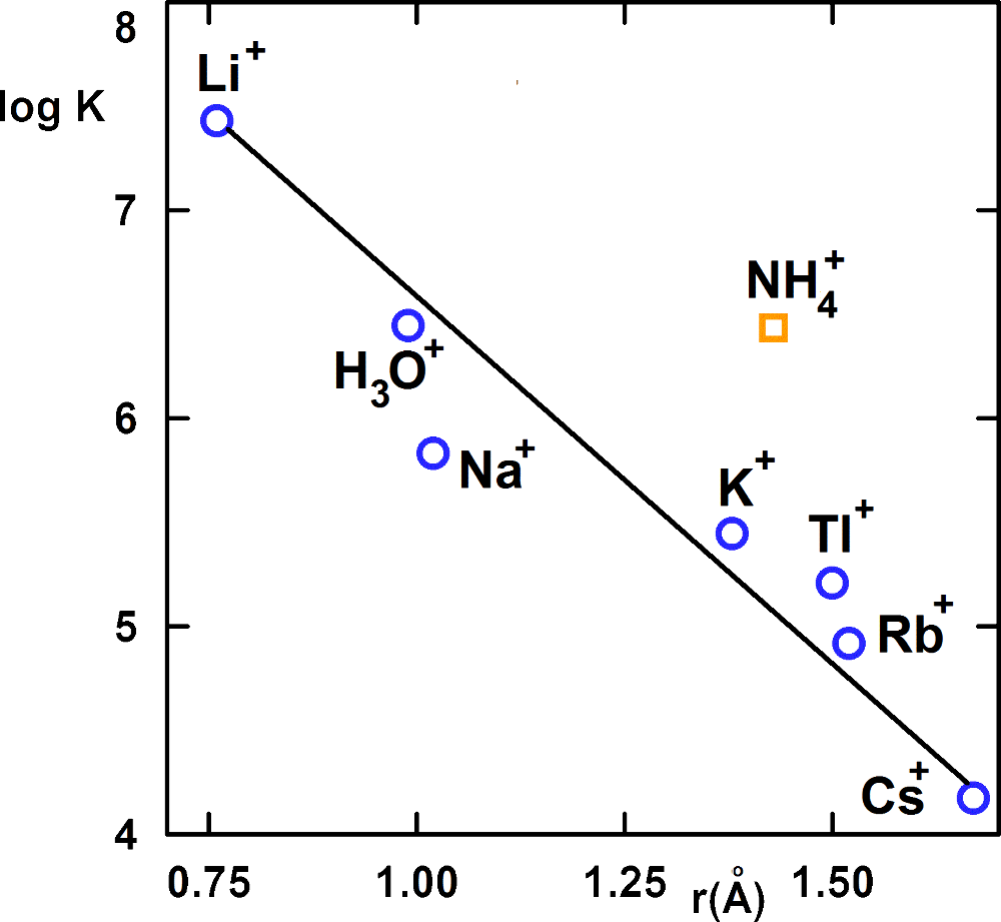
Logarithmic dependence of the stability constant of the **1·**M^+^ complex cation (M^+^ = Li^+^, Na^+^, K^+^, Rb^+^, Cs^+^, H_3_O^+^, NH_4_^+^, Tl^+^; **1** = enniatin B) in nitrobenzene saturated with water, log *K*_nb_(**1·**M^+^), on the crystallographic radius of the cation M^+^, r­(Å).

### 3.2 Quantum Mechanical Calculations

All quantum chemical calculations were performed using the Gaussian16 program package­(G16).^20^ As a computational method, density functional theory (DFT) was used with the D3 version of Grimme’s dispersion correction with the original D3 damping function.^21^ The M06 hybrid functional developed by Truhlar and Zhao^22^ was utilized with the Def2SVPP basis set by Ahlrichs and co-workers.^23^ Since all reactions proceeded in the solvent nitrobenzene, the effects of this solvent were considered based on the charge-density-based solvation model (SMD), a variant of the Integral-Equation-Formalism Polarizable Continuum Model (IEFPCM) developed by Truhlar and co-workers.^22^ Nitrobenzene was characterized by the following parameters: the dielectric constant (Eps) = 34.809; the dynamic or optical dielectric constant to infinity (EpsInf) = 2.421; H bond Acidity = 0.0; H bond Basicity = 0.28; Surface Tension At Interface = 57.54; Carbon Aromaticity = 0.667; Electronegative Halogenicity = 0.0.

Geometry optimization was carried out with an energy convergence criterion of 10^–9^ Hartree, and the ultrafine integration grid, as implemented in G16, was employed. As a matter of course, after the optimization procedure found a minimum, the respective vibrational frequency calculations were processed to confirm that the converged structure is a minimum on a particular potential energy surface. In the model calculations, we optimized the molecular geometries of the parent enniatin B ligand (**1**), its complex with H_3_O^+^ cation, and its complex with Na^+^ cation, similarly as in our previous papers.^10,12,14,15^ The optimized structure of the free ligand **1** [M06-D3/Def2SVPP in nitrobenzene] is illustrated in Figure 2, and optimized coordinates can be found in the Supporting Information.

**Figure 2:**
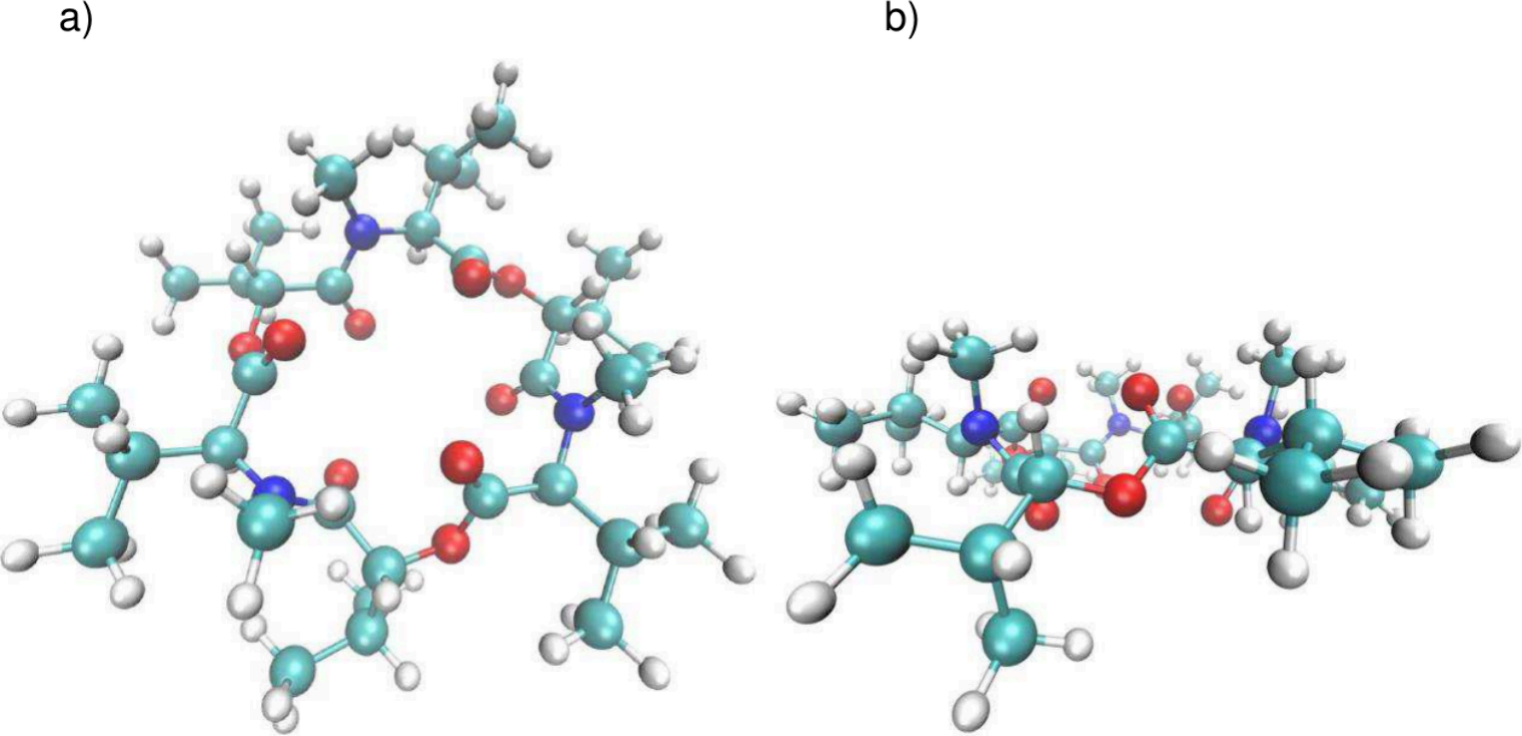
Two projections of the DFT-optimized structure of free ligand **1** [M06D3/Def2SVPP in nitrobenzene]: (a) top view and (b) side view.

To obtain the most probable structure of the **1·**H_3_O^+^ cationic complex with the lowest potential energy, six different initial mutual positions of ligand **1** and the H_3_O^+^ cation have been investigated. These positions resulted in three different conformers with local minima on the potential surface. The optimized structures with the energy differences relative to the zero point energy of the lowest structure are displayed in Figure 3, and optimized coordinates can be found in the Supporting Information.

**Figure 3:**
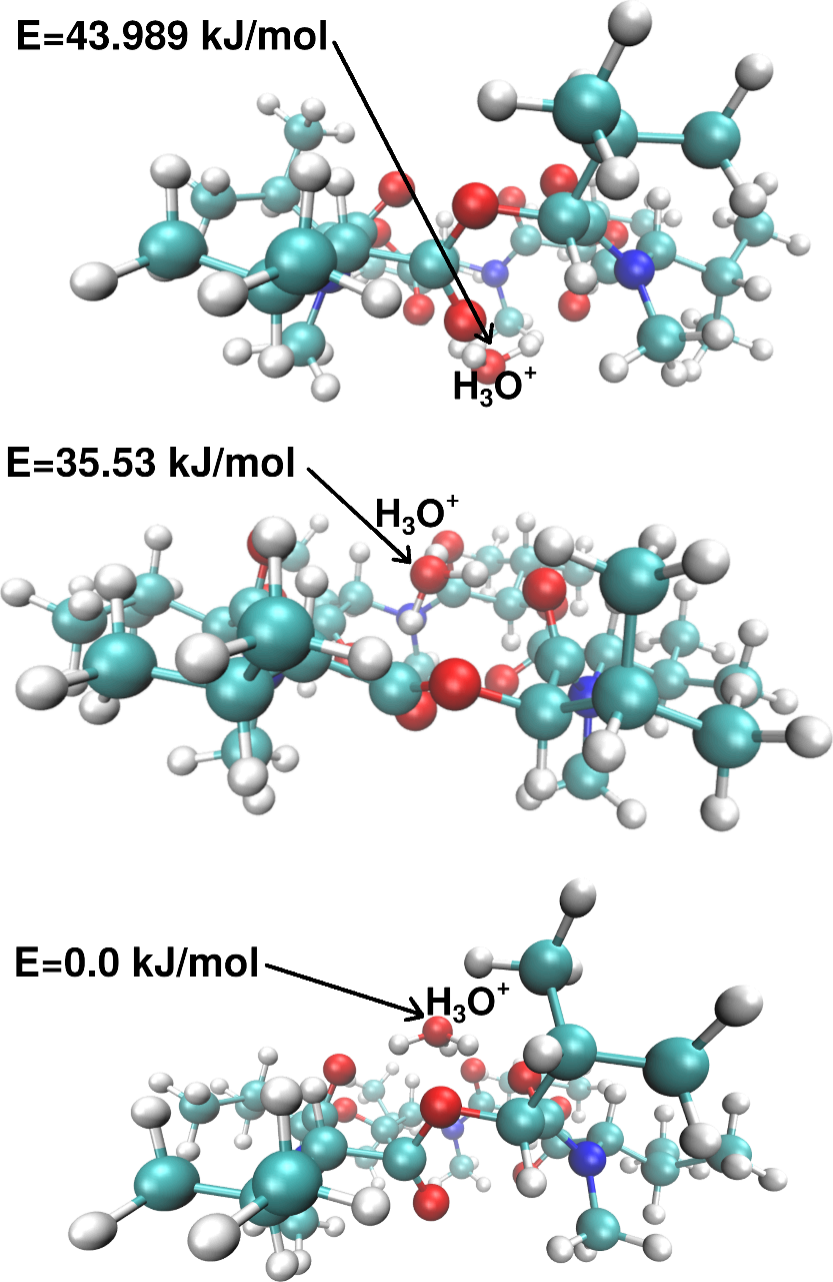
Optimized structure of the **1·**H_3_O^+^ complex of three different comforters [M06D3/Def2SVPP in nitrobenzene]. Energies are taken relative to the zero point energy of the lowest structure.

In Figure 4, the energetically lowest structure obtained by the full DFT-optimization of the **1·**H_3_O^+^ complex is depicted together with the lengths of the corresponding hydrogen bonds (in Å). It is clear that complexation with the H_3_O^+^ cation changes the overall shape of the parent ligand **1** only slightly. In the resulting **1·**H_3_O^+^ cationic complex species, which is most energetically favored, the “central” cation H_3_O^+^ is bound by three relatively strong hydrogen bonds to the corresponding three carbonyl oxygen atoms (1.52, 1.54, and 1.55 Å) of the parent **1** (see Figure 4). This structure is similar to that of the H_3_O^+^ beauvericin complex.^12^

**Figure 4:**
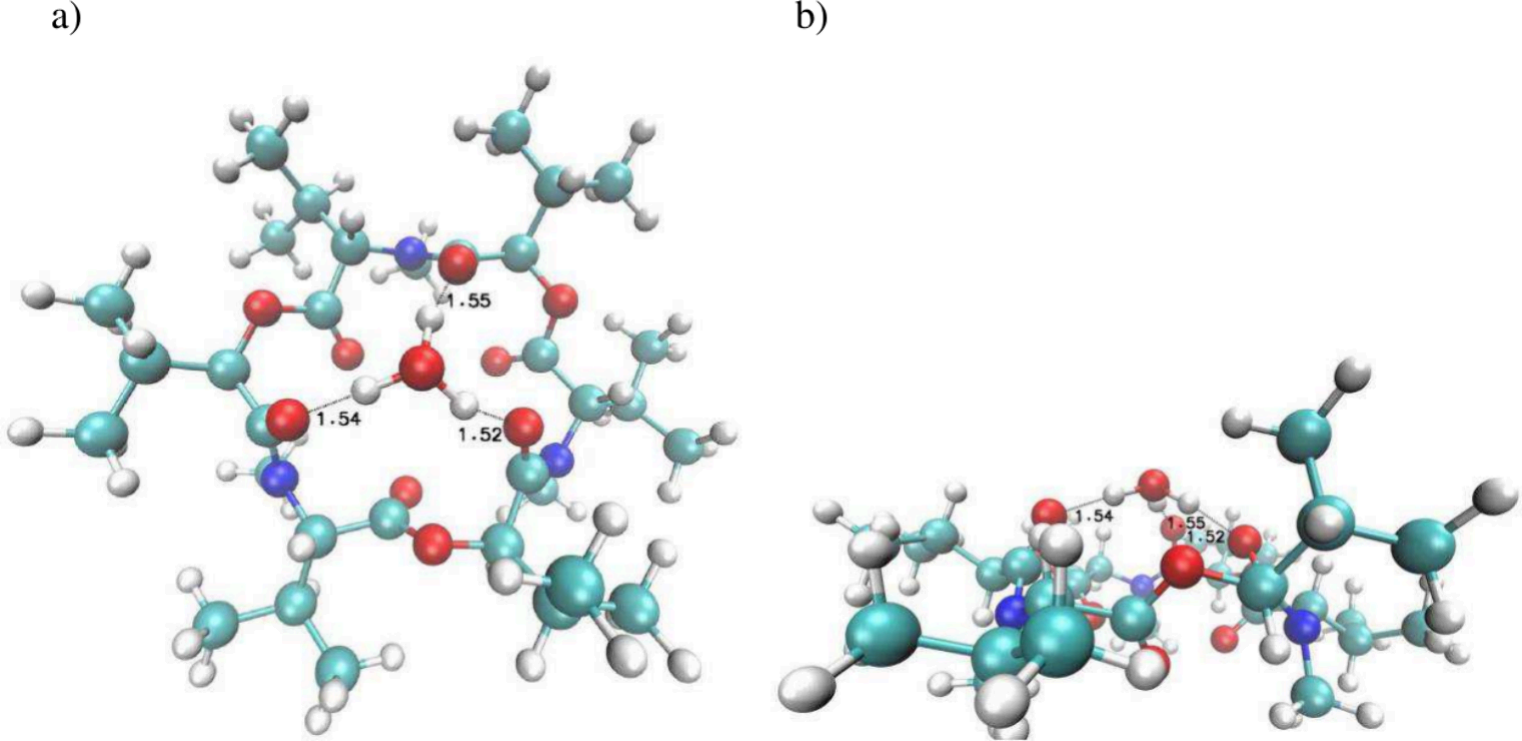
Two projections of the DFT-optimized structure of the energetically lowest **1·**H_3_O^+^ complex [M06-D3/Def2SVPP in nitrobenzene]: (a) top view and (b) side view. Hydrogen bond lengths of H_3_O^+^ to the respective three carbonyl oxygens of **1** are 1.52, 1.54, and 1.55Å.

Since this study is focused on the enniatin B protonation conducted by changing inner ions from Na^+^ to H_3_O^+^, the **1·**Na^+^ structure has also been calculated. The resulting optimized complex is displayed in Figure 5. The Na^+^ cation is held in the ligand cavity probably by ion bonding at the length of 2.3 Å from the respective three carbonyl oxygens.

**Figure 5:**
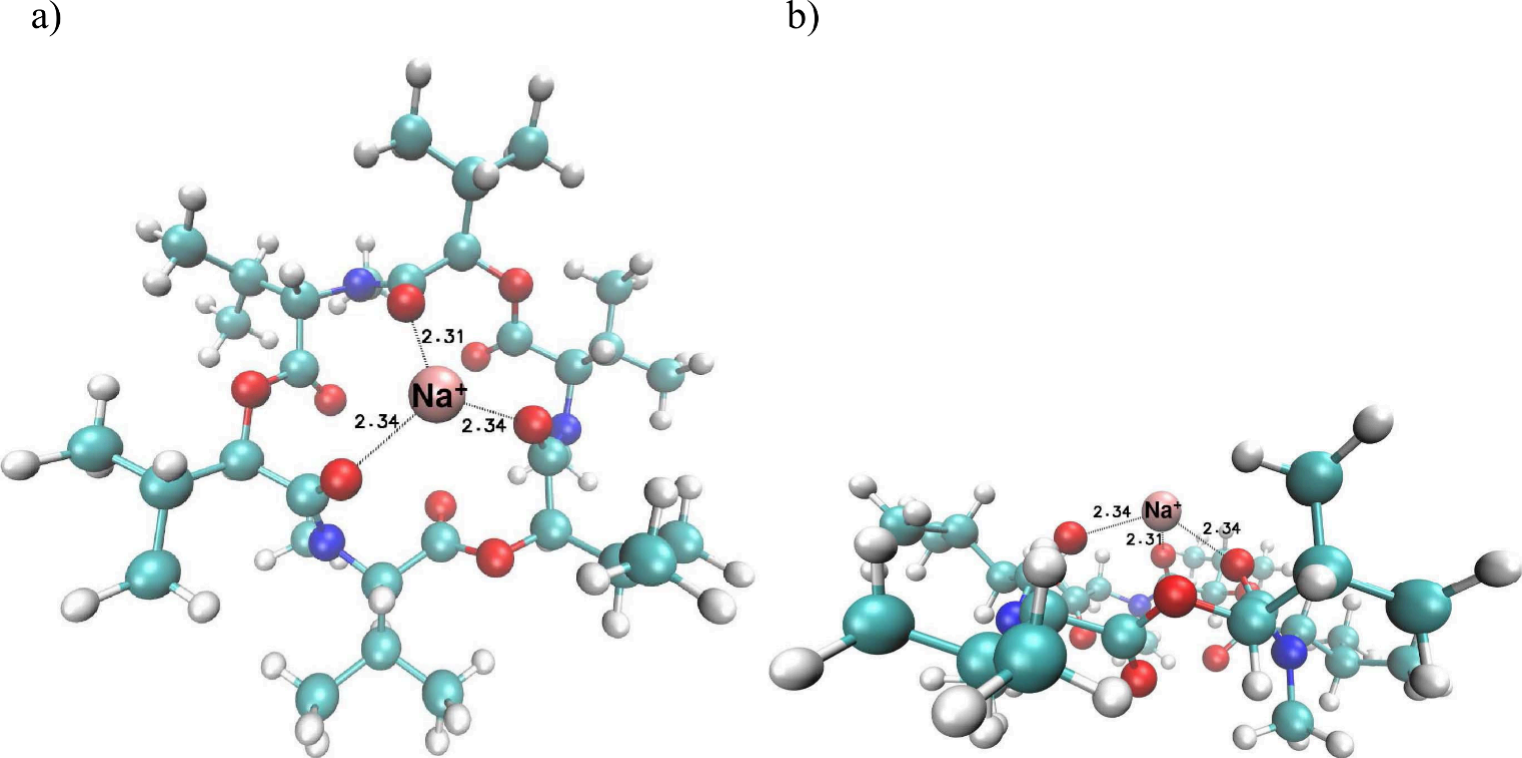
Two projections of the DFT-optimized structure of the **1·**Na^+^ complex [M06D3/Def2SVPP in nitrobenzene]: (a) top view and (b) side view. Bond lengths of Na^+^ to the respective three carbonyl oxygens of **1** are 2.31, 2.31, and 2.34 Å.

In our previous work^9^ we dealt with the complex of enniatin B with the ammonium cation. The structure of the NH4^+^–enniatin B complex is analogical to the structure of the H_3_O^+^ enniatin B complex; only three hydrogen atoms of ammonium are bonded to three oxygen atoms of enniatin B.

The calculated structures of the complexes of other univalent cations, the stability constants of which with enniatin are summarized in Table 4, are depicted in Figure 6.

**Figure 6:**
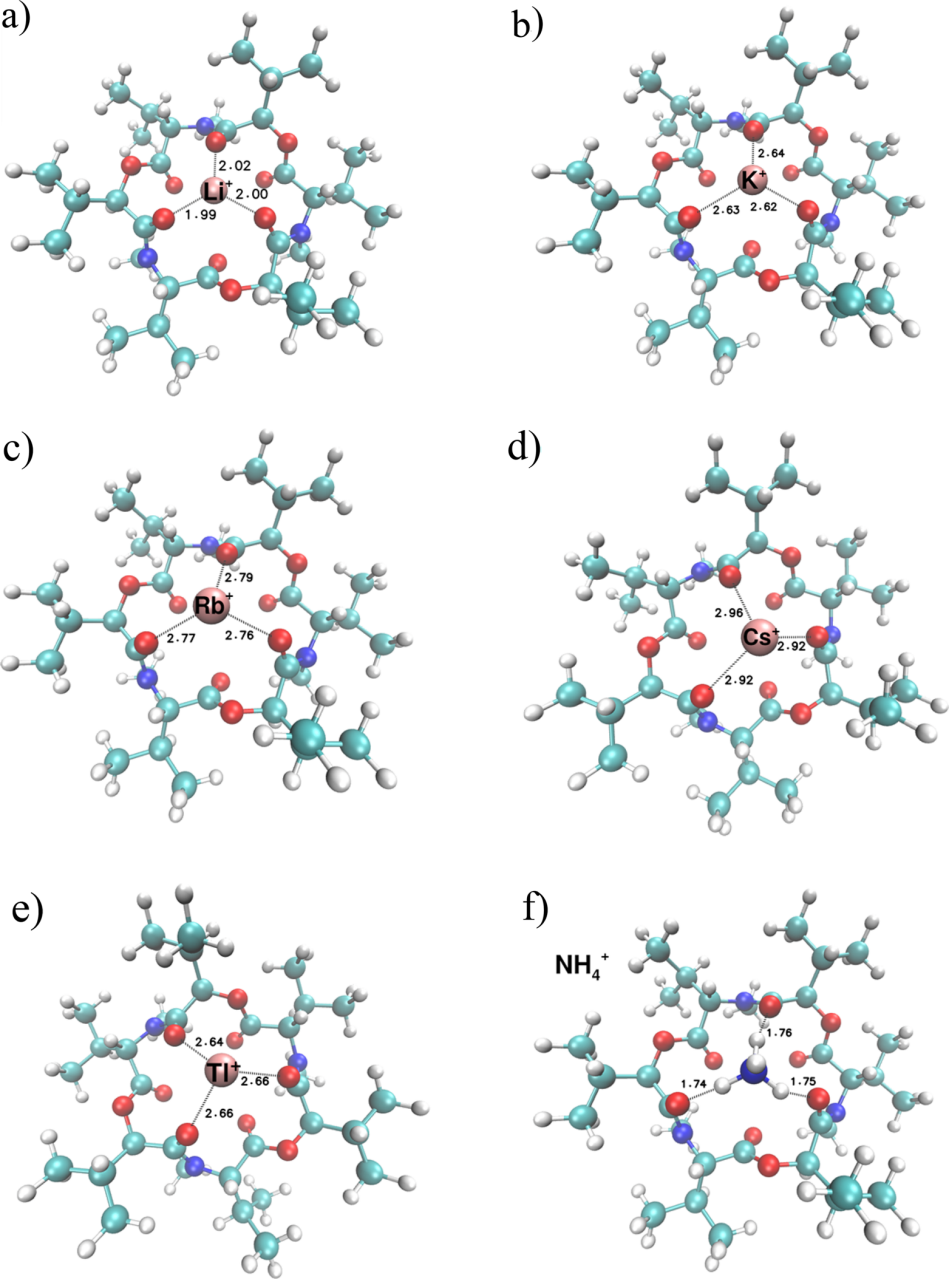
Calculated structures of complexes of univalent cations with Enniatin B. a) Li^+^, b) K^+^, c) Rb^+^, d) Cs^+^, e) Tl^+^ and f) NH_4_^+^; distances/Å.

It is clear from Figure 6 that the structures of the complexes of the univalent metal cation with enniatin B are similar. The cation is bonded by three bonds to three oxygens of the ligand molecule. The interaction energies and bond lengths are summarized in Table 5.

**Table 5:** Interaction Energies and Bond Lengths in the Complexes of Enniatin B with Some Univalent Cations

Ion	*E* (int) kJ/mol	r (Å)	Bond Lengths (Å)
Li^+^	80.31	0.76	1.99, 2.00, 2.02
Na^+^	82.19	1.02	2.31, 2.34, 2.34
K^+^	83.12	1.38	2.62, 2.63, 2.64
Rb^+^	78.30	1.52	2.76, 2.77, 2.79
Cs^+^	77.31	1.67	2.92, 2.92, 2.96
Tl^+^	118.92	1.50	2.64, 2.66, 2.66
NH_4_^+^	105.96	1.43	1.74, 1.75, 1.76
H_3_O^+^	196.81	0.99	1.52, 1.54, 1.55

Finally, the interaction energy, *E*(int), of complex **1·**H_3_O^+^ has been calculated with the base set superposition error correction (BSSE). The 7-point counterpoise method, as implemented in the Gaussian16 package program, was used. The results for different solvents and different molecules are collected in Table 6. The *E*(int) in nitrobenzene was found to be 196.8 kJ/mol, which confirms the formation of cationic complex **1·**H_3_O^+^ as well. The calculated interaction energy in vacuum is 468.98 kJ/mol.

**Table 6:** Interaction Energy, *E*(int) in kJ/mol, between Enniatin B and the Considered Species in the Complexes **1·**H_3_O^+^, **1·**Na^+^, and **1·**H_2_O Calculated by M06-D3/Def2SVPP in Nitrobenzene, Water, And Vacuum

Solution/Complex	**1**·H_3_O^+^	**1**·Na^+^	**1**·H_2_O
nitrobenzene	196.81	82.19	58.00
water	191.17	50.68	57.49
vacuum	468.98	409.15	61.17

The interaction energy of enniatin B calculated in the previous study in vacuum with ammonium is lower (305.5 kJ/mol) and the bond length is higher.^9^ On the other hand, the interaction energy of H_3_O^+^ cation with beauvericin (see Scheme 2), which structure is analogous to that of enniatin B, *E*(int) = 440.9 kJ/mol, is similar to that of enniatin B.^12^ The conformation of the H_3_O^+^ complex of beauvericin is the same as that of its complex with enniatin B, and even the bond lengths are almost the same, 1.55, 1.55, and 1.55 Å. This is caused by the fact that the structure of the inner cavity of both ligands is identical.

**Scheme 2:**
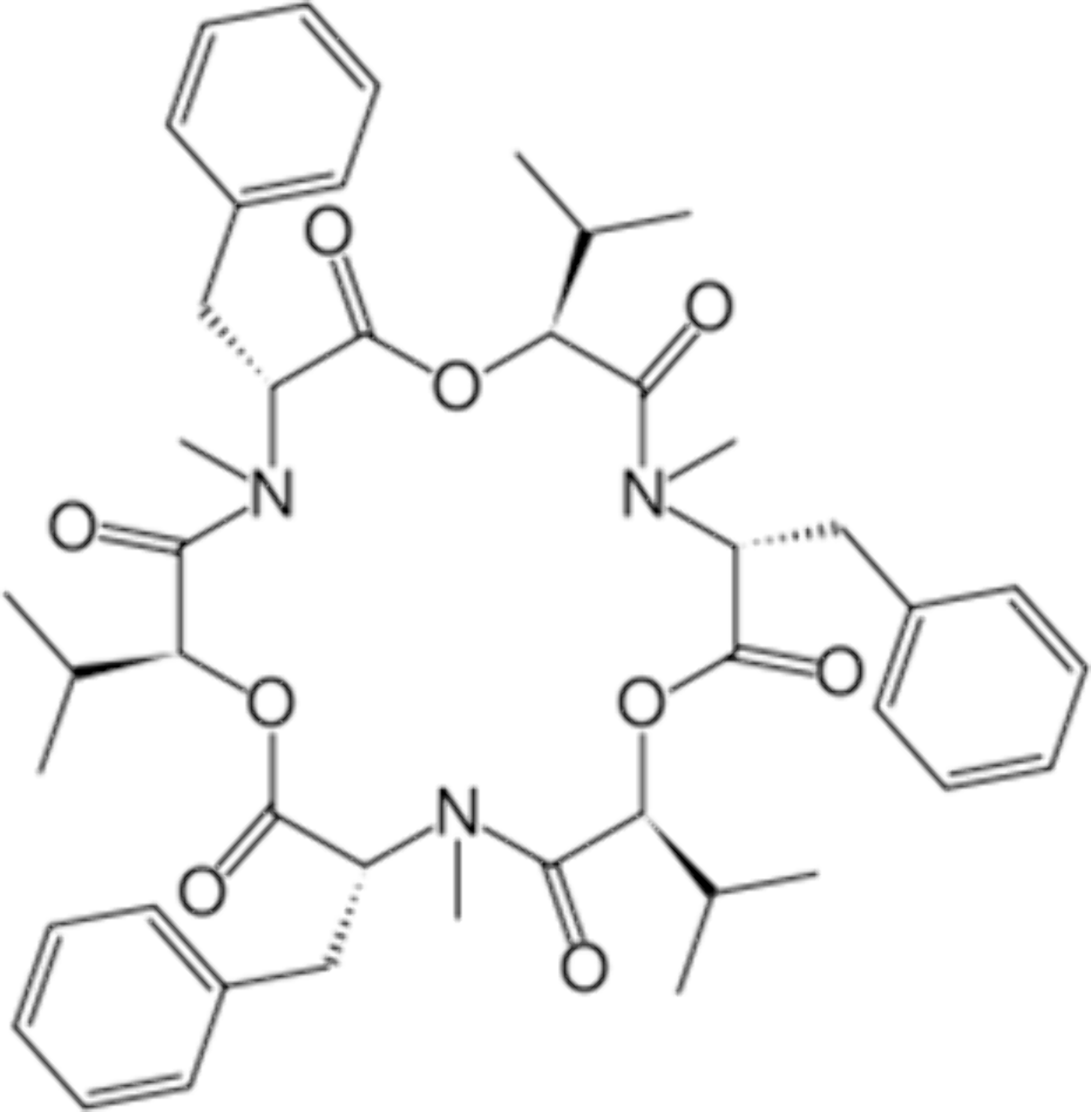
Structural Formula of Beauvericin

The structure of another cyclic natural ionophore, antamanide, is significantly different from that of enniatin B. However, the H_3_O^+^ cation is also located in the cavity of the ionophore and is bonded to its oxygens.^14^

## 4 Conclusions

Quantum mechanical calculations [M06-D3/Def2SVPP in nitrobenzene], combined with experimental extraction in a biphasic water–nitrobenzene system, have proven to be effective for studying noncovalent interactions between the H_3_O^+^ cation and the natural ionophore enniatin B (**1**). The stability constant of cationic complex **1**·H_3_O^+^ in water-saturated nitrobenzene was determined from extraction experiments to be log β (**1·**H_3_O^+^) = 6.4 ± 0.2 at 25 °C. Additionally, DFT calculations predicted the most likely structure of the **1·**H_3_O^+^ complex. In this structure, the central H_3_O^+^ cation is stabilized by three hydrogen bonds to the carbonyl oxygen atoms of the enniatin B ligand. This study provides valuable insights into the binding behavior of enniatin B and represents a noteworthy contribution to the field of supramolecular chemistry.

## Supplementary Material



## Notes

Supplementary data contain Cartesian coordinates of all optimized structures mentioned in the text. (PDF)

## References

[1] Gaumann E., Roth S., Ettlinger L., Plattner P. A., Nager U. (1947). Enniatin,
ein neues, gegen Mykobakterien wirksames Antibiotikum. [Enniatin,
a new antibiotic that works against mycobacteria]. Experientia.

[2] Grove J., Pople M. (1980). The insecticidal activity of beauvericin and the enniatin complex. Mycopathologia.

[3] Prosperini A., Berrada H., Ruiz M. J., Caloni F., Coccini T., Spicer L. J., Perego M. C., Lafranconi A. (2017). A Review of
the Mycotoxin Enniatin B. Front. Public Health.

[4] Lifson S., Felder C. E., Shanzer A. J. (1984). Enniatin
B and Valinomycin as Ion
Carriers: An Empirical Force Field Analysis. Biomol. Struct. Dyn..

[5] Ovchinnikov Y. A., Ivanov V. T., Evstratov A. V., Mikhaleva I. I., Bystrov V. F., Portnova S. L., Balashova T. A., Meshcheryakova E. N., Tulchinsky V. M. (1974). The enniatin ionophores. Conformation
and ion binding properties. Int. J. Pept. Protein
Res..

[6] Kamyar M., Rawnduzi P., Studenik C. R., Kouri K., Lemmens-Gruber R. (2004). Investigation
of the electrophysiological properties of enniatins. Arch. Biochem. Biophys..

[7] Ivanov V. T., Evstratov A. V., Sumskaya L. V., Melnik E. I., Chumburidze T. S., Portnova S. L., Balashova T. A., Ovchinnikov Y. A. (1973). Sandwich
complexes as a functional form of the enniatin ionophores. FEBS Lett..

[8] Makrlík E., Böhm S., Vaňura P., Raich I. (2014). Extraction and DFT
study on interaction of the cesium cation with enniatin B. J. Mol. Struct..

[9] Makrlík E., Böhm S., Vaňura P., Trnka L. (2015). Experimental and theoretical
study on complexation of the ammonium cation with enniatin B. J. Mol. Liq..

[10] Makrlík E., Böhm S., Vaňura P. (2016). Complexation
of the potassium cation
with enniatin B: an experimental and theoretical study. Monatsh. Chem..

[11] Makrlík E., Böhm S., Vaňura P. (2006). Experimental
Evidence for a Valinomycin
- Proton Complex. Monatsh. Chem..

[12] Makrlík E., Toman P., Vaňura P. (2012). A combined
experimental and DFT study
on the complexation of H_3_O^+^ with beauvericin. Monatsh. Chem..

[13] Makrlík E., Vaňura P. (2013). Synergistic extraction of some univalent
cations into
nitrobenzene by using sodium dicarbollylcobaltate and nonactin. J. Radioanal. Nucl. Chem..

[14] Makrlík E., Böhm S., Vaňura P., Ruzza P. (2014). Protonation of antamanide:
Experimental and theoretical study. J. Mol.
Liq..

[15] Toman P., Makrlík E., Vaňura P., Kašička V., Rathore R. (2010). A combined extraction and DFT study on the complexation
of H_3_O^+^ with a hexaarylbenzene-based receptor. Monatsh. Chem..

[16] Hawthorne M. F., Young D. C., Andrews T. D., Howe D. V., Pilling R. L., Pitts A. D., Reintjes M., Warren L. F., Wegner P. A. (1968). π-Dicarbollyl derivatives of
the transition metals.
Metallocene analogs. J. Am. Chem. Soc..

[17] Makrlík E., Vaňura P. (1985). Applications
of the Dicarbollylcobaltate­(III) anion
in the Water/Nitrobenzene Extraction System. Talanta.

[18] Makrlík E., Vaňura P. (1998). Extraction of sodium picrate into nitrobenzene in the
presence of valinomycin. ACH Models Chem..

[19] Rais J. (1971). Individual
extraction constants of univalent ions in the system water-nitrobenzene. Collect. Czech. Chem. Commun..

[20] Frisch, M. J. ; Gaussian ∼ 16 Revision B.01. 2016; Gaussian Inc.: Wallingford, CT.

[21] Grimme S., Antony J., Ehrlich S., Krieg H. A. (2010). A consistent and
accurate ab initio parametrization of density functional dispersion
correction (DFT-D) for the 94 elements H-Pu. J. Chem. Phys..

[22] Zhao Y., Truhlar D. G. (2008). The M06 suite of
density functionals for main group
thermochemistry, thermochemical kinetics, noncovalent interactions,
excited states, and transition elements: two new functionals and systematic
testing of four M06-class functionals and 12 other functionals. Theor. Chem. Acc..

[23] Pritchard B. P., Altarawy D., Didier B., Gibson T. D., Windus T. L. (2019). New Basis
Set Exchange: An Open, Up-to-Date Resource for the Molecular Sciences
Community. J. Chem. Inf. Model..

