# Supplementary material for: Protonation of Enniatin B in Nitrobenzene: Experimental and Theoretical Study with Respect to Other Univalent Cations
Source: ACS Omega. 2025 Sep 17;10(38):43532–9. doi: 10.1021/acsomega.5c02470 (PMC12489657; doi:10.1021/acsomega.5c02470)
Supplement: Supplementary file 1 [file ao5c02470_si_001.pdf]

Supplementary data

Protonation of enniatin B in nitrobenzene: Experimental and theoretical study with respect to other univalent cations

Petr Vaňura, Stanislav Böhm, and Tereza Uhlíková*

University of Chemistry and Technology, Technická 5, Prague, Prague 6 166 28, Czech Republic

* E-mail: petr.vanura@vscht.cz

Table S1. Cartesian coordinates (in Å) for free ligand **1** (**1** = enniatin B, $C_{33}H_{57}N_3O_9$)

102 atoms

N	2.694410	-2.055478	0.494460
C	0.382100	-2.789461	0.409961
O	0.038560	-2.112642	1.348240
C	3.114802	-1.930695	1.883817
C	1.819162	-3.145312	0.083083
C	2.237758	-4.519187	0.642258
C	1.319994	-5.635352	0.156982
C	3.678180	-4.812095	0.245273
O	-0.471137	-3.343437	-0.449099
C	2.791257	-1.020427	-0.388688
O	2.146789	-0.968851	-1.424299
C	-1.857239	-3.234121	-0.125908
C	-2.620107	-4.187648	-1.050383
C	-2.402079	-3.918477	-2.531567
C	-4.102684	-4.197207	-0.705495
C	3.839980	0.055687	-0.119219
C	-2.311399	-1.789263	-0.296695
O	3.268060	1.336201	-0.397002
C	5.026496	-0.109205	-1.066139
N	-3.187166	-1.289386	0.625405
O	-1.965084	-1.156301	-1.281380
C	2.252211	1.717658	0.375927
C	5.610643	-1.512207	-0.982238
C	6.086618	0.942283	-0.775364
C	-3.219233	-1.780875	1.997320
C	-3.769224	0.000412	0.271109
O	1.796065	1.043862	1.265735
C	1.785861	3.110087	0.012878
C	-2.748922	1.097369	0.503331
C	-5.111560	0.290244	0.962560
N	0.404182	3.294387	0.424081
C	2.765390	4.202708	0.517580
O	-1.976558	1.125320	1.431037
O	-2.822207	2.046200	-0.427386
C	-5.675195	1.639309	0.534232
C	-6.102442	-0.822007	0.648451
C	0.073240	3.784038	1.752210
C	-0.518507	2.752132	-0.419165
C	3.587818	3.799301	1.738305
C	3.688935	4.634124	-0.614333
C	-1.971858	3.181375	-0.259797
O	-0.207923	2.046927	-1.366681
C	-2.330242	4.176868	-1.363198
C	-1.392176	5.377139	-1.350128
C	-3.779951	4.618972	-1.235644
H	1.884469	-3.210441	-1.018664

H	2.167578	-4.493034	1.748506
H	2.594264	-2.684353	2.491614
H	2.827483	-0.944420	2.283419
H	4.203342	-2.082174	2.013890
H	-1.988496	-3.581364	0.913137
H	-2.200542	-5.189054	-0.816838
H	4.196424	0.053949	0.927419
H	4.622258	0.048618	-2.087231
H	-3.963470	-0.023412	-0.817210
H	-3.652746	-1.012734	2.653114
H	-2.195320	-1.973194	2.359371
H	-3.827992	-2.698838	2.104688
H	1.781512	3.143269	-1.091047
H	-4.949364	0.336410	2.058428
H	2.136405	5.074390	0.788112
H	-0.711397	3.154558	2.202858
H	-0.253572	4.841715	1.752352
H	0.957004	3.702720	2.401882
H	-2.181504	3.631574	0.728232
H	-2.197924	3.629564	-2.319046
H	1.714063	-6.612104	0.492136
H	1.263713	-5.653714	-0.949404
H	0.289802	-5.544491	0.545505
H	3.991904	-5.801286	0.625928
H	4.387675	-4.060836	0.635197
H	3.776092	-4.827251	-0.858856
H	-2.863565	-4.727520	-3.127916
H	-1.328711	-3.874847	-2.786911
H	-2.863593	-2.962656	-2.840430
H	-4.625221	-4.979371	-1.285532
H	-4.580684	-3.228482	-0.954665
H	-4.281751	-4.401227	0.367720
H	6.499466	-1.593682	-1.633751
H	4.892587	-2.289428	-1.304508
H	5.936368	-1.750601	0.050575
H	6.935483	0.843781	-1.476732
H	6.483158	0.821015	0.252861
H	5.688117	1.968243	-0.868528
H	-6.675168	1.786597	0.981199
H	-5.785035	1.692993	-0.567150
H	-5.043279	2.488457	0.853613
H	-5.757358	-1.811359	0.998138
H	-6.265742	-0.890586	-0.445835
H	-7.079442	-0.618200	1.123687
H	4.303893	2.991334	1.485830
H	2.973406	3.444163	2.585583
H	4.183232	4.659130	2.095284
H	4.295838	3.780066	-0.972299
H	4.383325	5.424984	-0.275379
H	3.115716	5.026364	-1.474946

H	-1.699859	6.105889	-2.121731
H	-0.340390	5.103040	-1.557745
H	-1.424865	5.898774	-0.372351
H	-4.038928	5.334371	-2.037836
H	-3.949153	5.126419	-0.264308
H	-4.477754	3.766366	-1.302265

Table S2. Cartesian coordinates (in Å) for **1**·H_3O^+ complex

106 atoms

N	-3.073068	-1.577576	-0.635743
C	-0.898721	-2.639127	-0.459331
O	-0.414308	-2.163317	-1.454423
C	-3.429158	-1.374821	-2.036807
C	-2.381785	-2.791055	-0.202340
C	-2.930818	-4.089208	-0.816551
C	-2.163598	-5.299506	-0.296510
C	-4.414640	-4.214337	-0.502915
O	-0.174348	-3.145192	0.544426
C	-3.087225	-0.569711	0.254922
O	-2.553478	-0.661069	1.366952
C	1.208192	-3.365103	0.271773
C	1.758920	-4.268040	1.383405
C	1.562685	-3.715469	2.787493
C	3.218211	-4.611418	1.120663
C	-3.861643	0.701546	-0.053387
C	1.945580	-2.042688	0.219484
O	-3.050486	1.822778	0.309660
C	-5.136332	0.766794	0.783981
N	2.932352	-1.846164	-0.677041
O	1.721945	-1.192907	1.091164
C	-1.878629	1.927058	-0.315973
C	-5.995693	-0.470485	0.560476
C	-5.912682	2.036699	0.471781
C	2.950757	-2.450944	-2.003013
C	3.774088	-0.677027	-0.400985
O	-1.448620	1.090496	-1.070828
C	-1.169718	3.208700	0.060129
C	2.963793	0.580841	-0.648832
C	5.117626	-0.705944	-1.146399
N	0.201219	3.168082	-0.427856
C	-1.942767	4.494942	-0.343349
O	2.149906	0.712729	-1.529468
O	3.262016	1.532795	0.233409
C	5.871057	0.608139	-0.986638
C	5.963901	-1.861992	-0.628487
C	0.495870	3.572717	-1.797485
C	1.113118	2.587720	0.372111

C	-2.900854	4.324887	-1.519373
C	-2.679378	5.062728	0.862762
C	2.591809	2.782518	0.076503
O	0.814151	2.001274	1.417619
C	3.189927	3.756074	1.091978
C	2.434270	5.079067	1.089778
C	4.669000	3.970129	0.810561
H	-2.522728	-2.865457	0.889925
H	-2.787560	-4.056858	-1.915355
O	-0.223043	-0.158756	2.285379
H	-3.164041	-2.271266	-2.612467
H	-2.859954	-0.528384	-2.456538
H	-4.512144	-1.201443	-2.163951
H	1.283663	-3.912927	-0.682035
H	1.155517	-5.195005	1.289521
H	-4.109558	0.796060	-1.126048
H	-4.805659	0.788812	1.842546
H	4.002729	-0.686623	0.681230
H	2.981020	-1.650608	-2.761290
H	2.022209	-3.008819	-2.180208
H	3.814082	-3.123491	-2.148616
H	-1.102739	3.193469	1.163460
H	4.923516	-0.847042	-2.227497
H	-1.162458	5.227675	-0.630776
H	1.170678	2.839970	-2.266718
H	0.935361	4.585276	-1.854736
H	-0.436487	3.569363	-2.379826
H	2.776718	3.156226	-0.946423
H	3.071563	3.275074	2.084153
H	-2.631176	-6.227108	-0.672344
H	0.060423	0.778351	1.988739
H	-2.175765	-5.336660	0.810549
H	-1.107442	-5.311546	-0.625496
H	-4.821091	-5.152477	-0.922038
H	-1.165860	-0.380406	1.961432
H	-5.008892	-3.378508	-0.914646
H	0.484978	-0.787092	1.877473
H	-4.575335	-4.234153	0.593443
H	1.830134	-4.490689	3.528686
H	0.514546	-3.419102	2.973443
H	2.208132	-2.837316	2.975181
H	3.558018	-5.390969	1.825732
H	3.874427	-3.729888	1.267799
H	3.381253	-4.993430	0.094350
H	-6.929270	-0.393319	1.145726
H	-5.490578	-1.404641	0.871856
H	-6.284178	-0.571528	-0.505389
H	-6.834896	2.083142	1.079045
H	-6.209962	2.060450	-0.595750
H	-5.323587	2.946160	0.684290

H	6.863819	0.527387	-1.464844
H	6.029791	0.850742	0.082416
H	5.347820	1.461563	-1.457445
H	5.455512	-2.839640	-0.711245
H	6.211087	-1.704133	0.440066
H	6.914260	-1.929225	-1.188509
H	-3.781758	3.718130	-1.234602
H	-2.435859	3.844617	-2.400333
H	-3.276660	5.311903	-1.843477
H	-3.422627	4.339139	1.248347
H	-3.217399	5.989150	0.590363
H	-1.979722	5.302566	1.685157
H	2.913001	5.788317	1.788423
H	1.378385	4.973350	1.405415
H	2.449400	5.546562	0.084639
H	5.105125	4.665516	1.550791
H	4.815886	4.412336	-0.195237
H	5.237966	3.025292	0.856444

Table S3. Cartesian coordinates (in Å) for **1**·Na^+ complex

103 atoms

N	-2.761058	-1.972535	-0.583156
C	-0.477430	-2.782997	-0.507777
O	-0.099261	-2.137953	-1.454606
C	-3.261950	-1.827529	-1.946139
C	-1.924215	-3.107097	-0.203232
C	-2.375104	-4.447649	-0.809658
C	-1.456437	-5.588943	-0.388679
C	-3.808999	-4.739052	-0.388532
O	0.343329	-3.321802	0.393289
C	-2.750074	-0.935886	0.287843
O	-2.007892	-0.907521	1.267732
C	1.734388	-3.249154	0.094756
C	2.463583	-4.168100	1.081504
C	2.259554	-3.790962	2.540915
C	3.943224	-4.267652	0.740430
C	-3.786918	0.164564	0.115893
C	2.205042	-1.806430	0.195355
O	-3.177091	1.429677	0.369789
C	-4.911559	-0.001199	1.137960
N	3.149842	-1.383934	-0.681772
O	1.801295	-1.091879	1.109887
C	-2.185402	1.786362	-0.446182
C	-5.520805	-1.395031	1.075521
C	-5.972770	1.068016	0.926468
C	3.238871	-1.941170	-2.028369

C	3.768015	-0.104036	-0.339914
O	-1.763358	1.089477	-1.335027
C	-1.698333	3.180242	-0.124813
C	2.774738	1.013173	-0.582353
C	5.116770	0.149586	-1.031993
N	-0.306286	3.316774	-0.531677
C	-2.652422	4.270491	-0.678233
O	2.010419	1.062974	-1.514926
O	2.862608	1.951971	0.358308
C	5.698061	1.497502	-0.622798
C	6.091712	-0.969952	-0.693797
C	0.055100	3.853803	-1.835432
C	0.581902	2.711716	0.289077
C	-3.453137	3.840384	-1.903891
C	-3.594319	4.739839	0.423132
C	2.045874	3.107702	0.194332
O	0.224140	1.957936	1.193085
C	2.395027	4.068132	1.333730
C	1.478311	5.284504	1.341725
C	3.854335	4.487351	1.238845
H	-2.002838	-3.212274	0.894867
H	-2.325940	-4.377623	-1.914549
Na	-0.016766	-0.107148	2.203575
H	-2.793314	-2.586163	-2.588342
H	-2.974446	-0.843513	-2.350087
H	-4.358438	-1.953141	-2.012243
H	1.883187	-3.647352	-0.923367
H	1.997460	-5.161461	0.911636
H	-4.212282	0.181784	-0.904186
H	-4.442041	0.139858	2.133405
H	3.964875	-0.122722	0.748544
H	3.716760	-1.209432	-2.694191
H	2.228523	-2.128094	-2.427918
H	3.832074	-2.873523	-2.064455
H	-1.705270	3.259148	0.977017
H	4.957845	0.182143	-2.128549
H	-2.011150	5.130180	-0.955935
H	0.861577	3.248704	-2.278838
H	0.363719	4.915024	-1.793272
H	-0.808522	3.772405	-2.511549
H	2.292445	3.579288	-0.774707
H	2.241014	3.497433	2.272783
H	-1.862573	-6.547085	-0.760250
H	-1.381765	-5.657428	0.714435
H	-0.432086	-5.488128	-0.792301
H	-4.155267	-5.693354	-0.825170
H	-4.515025	-3.949375	-0.702110
H	-3.872996	-4.828191	0.714405
H	2.678301	-4.581858	3.190079
H	1.190071	-3.675437	2.791967

H	2.775076	-2.844547	2.790621
H	4.430986	-5.022742	1.382836
H	4.464325	-3.304541	0.912768
H	4.111090	-4.566851	-0.311691
H	-6.360717	-1.471782	1.789123
H	-4.796449	-2.190947	1.332994
H	-5.926523	-1.611526	0.066598
H	-6.772496	0.975260	1.683764
H	-6.439989	0.959930	-0.072757
H	-5.553909	2.087260	0.999799
H	6.700266	1.622295	-1.070923
H	5.808237	1.566315	0.477586
H	5.080012	2.351260	-0.956951
H	5.739356	-1.962464	-1.026430
H	6.251597	-1.019543	0.401842
H	7.072022	-0.786329	-1.169869
H	-4.188093	3.052563	-1.640634
H	-2.826440	3.448094	-2.725257
H	-4.027261	4.697100	-2.300313
H	-4.220434	3.903582	0.789454
H	-4.270996	5.528920	0.047281
H	-3.035473	5.149135	1.285068
H	1.790629	5.984598	2.137303
H	0.419062	5.026398	1.531183
H	1.532893	5.833936	0.380604
H	4.115033	5.168277	2.069624
H	4.043926	5.026473	0.288878
H	4.536027	3.620355	1.282856
